# Associations of Family Functioning and Social Support With Psychopathology in Children of Mentally Ill Parents: Multilevel Analyses From Different Rating Perspectives

**DOI:** 10.3389/fpsyg.2021.705400

**Published:** 2021-09-14

**Authors:** Marlit Sell, Claus Barkmann, Bonnie Adema, Anne Daubmann, Reinhold Kilian, Maja Stiawa, Mareike Busmann, Sibylle M. Winter, Martin Lambert, Karl Wegscheider, Silke Wiegand-Grefe

**Affiliations:** ^1^Department of Child and Adolescent Psychiatry and Psychotherapy, University Medical Center Hamburg-Eppendorf, Hamburg, Germany; ^2^Department of Medical Biometry and Epidemiology, University Medical Center Hamburg-Eppendorf, Hamburg, Germany; ^3^Department of Psychiatry and Psychotherapy II, Ulm University, Ulm, Germany; ^4^Department of Child and Adolescent Psychiatry, Psychosomatics and Psychotherapy, Charité – Universitätsmedizin Berlin, Corporate Member of Freie Universität Berlin, Humboldt-Universität zu Berlin, and Berlin Institute of Health (BIH), Campus Virchow-Klinikum, Berlin, Germany; ^5^Department of Psychiatry and Psychotherapy, University Medical Center Hamburg-Eppendorf, Hamburg, Germany

**Keywords:** parental mental illness, child psychopathology, family functioning, social support, multi-informant data

## Abstract

Offspring of mentally ill parents is at heightened risk for psychological symptoms. The identification of environmental factors that predict their mental health is crucial for the development of preventive and therapeutic measures. In the current study, we addressed the combined role of family functioning and social support by taking mentally ill patients’, their partners’, and children’s perspectives into account. The cross-sectional sample included *n*=195 families (195 patients, 127 partners, and 295 children). Family members completed questionnaires related to family functioning, social support as well as parental and child psychopathology. We conducted multilevel analyses to investigate the associations with internalizing and externalizing problems in children. Family functioning and social support were significantly associated with child internalizing and externalizing problems. However, results varied depending on the rating perspective. We found significant interaction effects of family functioning and social support on child psychopathology. The findings point to the importance of family functioning and social support as potential targets for interventions. Findings should be replicated in future longitudinal studies.

## Introduction

Parental mental illness is highly prevalent, with recent evidence from European cohorts suggesting that approximately 20% of children live with a mentally ill parent (Plass-Christl et al., 2017; Abel et al., 2019). There is a large body of research showing that children of mentally ill parents are at increased risk of developing mental health problems themselves (Ford et al., 2007; Siegenthaler et al., 2012; Rasic et al., 2013; van Santvoort et al., 2015). Parental psychopathology is also linked to poor cognitive and social outcomes in children such as lower attainment in communication, language, and literacy as well as personal, social, and emotional development (Mensah and Kiernan, 2010). For these children, the elevated risk for psychiatric disorders and related problems like poor social functioning and impaired physical health also persists into adulthood (Weissman et al., 2006). However, a substantial number of children exposed to parental mental illness do not develop adverse outcomes and it is important to consider aspects of risk and resilience (Grove et al., 2017). Both genetics and environmental factors play a role in the intergenerational transmission of psychopathology (Beardslee et al., 2011). Environmental factors are potentially modifiable and can be targeted in prevention and treatment (Burstein et al., 2012). Therefore, it is of great importance to identify environmental factors that predict mental health in children of mentally ill parents.

Among environmental factors, the relevance of family variables has been highlighted in the previous research (Rohde, 2013). One such family variable is family functioning, which is a multidimensional construct that indicates the collective functioning of a family (Daches et al., 2018; Wiegand-Grefe et al., 2019). Dimensions of family functioning include, for example, communication, affective involvement, and problem-solving within the family (Steinhauer et al., 1984; Miller et al., 2000). Parental mental illness was found to be associated with poor family functioning across a variety of disorders including major depression, bipolar disorder, and psychosis (Weinstock et al., 2006; Koutra et al., 2014). There is evidence that poor family functioning is also related to child mental health problems in families where a parent has a mental illness. Freed et al. (2015) found that higher family conflict and lower cohesion are associated with higher internalizing and externalizing problems in children of patients with a bipolar disorder. Several dimensions of family functioning and children’s internalizing as well as externalizing problems were also correlated in a sample of families affected by different parental mental disorders (Wiegand-Grefe et al., 2019). Family functioning was also found to be a mediator in the relationship between parental and child psychopathology in families with parental depression (Daches et al., 2018) as well as substance abuse (Burstein et al., 2012), which points at the importance of family functioning in the intergenerational transmission of mental illnesses.

Besides family factors, characteristics of the social environment such as social support are assumed to be important for the mental health of children with mentally ill parents (Van Santvoort et al., 2014). In the general population, there is an overall positive relationship between social support and well-being in children and adolescents (Chu et al., 2010). Hoefnagels et al. (2006) also found that social support predicts psychopathology in the offspring of psychiatric patients. Children of mentally ill parents often perceive a lack of social support and feel socially isolated (Hoefnagels et al., 2006; Reupert and Maybery, 2016). However, positive perceptions of social support are assumed to have a stress-buffering effect (Cohen and Wills, 1985). Resilient children of mentally ill parents have been shown to benefit from support outside the family (Werner, 1993), and by different family members, Tannenbaum and Forehand (1994) found father–child relationship to be a buffer for maternal depressive mood on child internalizing and externalizing problems. In families affected by a parental anxiety disorder, a positive sibling relationship was found to moderate the association between parental psychological distress and child adjustment (Keeton et al., 2015).

In a similar manner, child social support may also buffer against the negative effects of family dysfunction on child mental health. Dysfunctional family relationships represent a stressor for children and adolescents (Jiménez et al., 2019). This stressor may have a stronger impact on child adjustment for children with a lack of social support (Gauze et al., 1996; Daches et al., 2018; McLafferty et al., 2018). Although there is evidence indicating that family functioning and social support play important roles in the adverse effects of parental mental disorders on child mental health, few studies have considered these constructs simultaneously. For example, Plass et al. (2016) included family climate and social support as predictors of mental health in children of parents with mental health problems in their study. However, the authors did not consider a possible interaction effect between both constructs. Also, the analyses were limited to children’s self-ratings.

The importance of multi-informant data for the assessment of family variables and child mental health has been underlined by several researchers (Burt et al., 2005; Van Loon et al., 2014). Family members have shown divergent perspectives on family functioning (Weinstock et al., 2013; De Los Reyes et al., 2019). Also, there is significant variation in the association between several risk factors and child psychopathology across informants (Collishaw et al., 2009). Previous studies reveal that mentally ill parents’ reports of child psychopathology may be biased due to their mental illness (De Los Reyes and Kazdin, 2005; Maoz et al., 2014). This makes multiple sources of information especially relevant in the context of parental mental illness.

Building on the findings of the previous research, we hypothesized that family functioning and social support will be associated with the extent of children’s internalizing and externalizing problems. We also hypothesize that the association between family functioning and internalizing as well as externalizing problems will be moderated by child social support. We took different perspectives into account (mentally ill parents, partners, and children) and looked at whether the associations were robust across informants.

## Materials and Methods

### Study Design

For the present analyses, a cross-sectional one-group design was used. Data from the baseline assessment of a multicenter randomized controlled trial were used (“Implementation and evaluation of a family-based intervention program for children of mentally ill parents: a randomized controlled multicenter trial”; for study protocol, see Wiegand-Grefe et al., 2021). The trial was conducted at seven clinical centers located in Germany and Switzerland. Recruitment and data collection were performed between 2014 and 2017. For study participation, one parent in the family (hereinafter also referred to as “patient”) had to meet the diagnostic criteria of a mental disorder according to ICD-10 rated by an attending clinician. Consent to participate in the study and sufficient knowledge of the German language by parents and children were required. Acute severe parental psychiatric symptoms requiring inpatient treatment were an exclusion criterion. For the current analyses, children were included if they were in the age range of 4–18years old due to the predefined age ranges of the administered questionnaires. Ratings of patients, their partners, and children (self-report from the age of 12years old onward) in standardized psychometric questionnaires were used. The study was approved by the Ethics Committee of the Chamber of Physicians in Hamburg, Germany.

### Sample

The overall trial sample consisted of *n*=216 families with 216 patients, 145 partners, and 338 children. The data of 29 children were excluded because they were outside the range of 4–18years and 14 removed due to complete or extensive (>30% of items) missing data for the baseline assessment. The resulting sample comprised 295 children from 195 families, including 195 patients and 127 partners. Of the families, 55.9% participated with one child, 35.9% with a pair of siblings, 6.7% with three siblings and 1.5% with four siblings. The mean age of patients was *M*=40.38years (*SD*=6.95), of partners *M*=40.60years (*SD*=6.53), and of children *M*=9.99years (*SD*=4.04). Further characteristics of the sample are displayed in Table 1. In our sample, the most prevalent parental mental illnesses were affective disorders (ICD-10, F30-F39), followed by personality disorders (ICD-10, F60-F69), and neurotic, stress-related, and somatoform disorders (ICD-10; F40-F48).

**Table 1:** Characteristics of patients, partners, and children.

	Patients (*N* =195)		Partners (*N* =127)		Children (*N* =295)	
	*n*	%	*n*	%	*n*	%
Gender (female)	146	74.9	47	37.0	154	52.2
Living with both parents					166	56.3
Marital status						
Married	108	55.4	95	74.8		
Unmarried	45	23.1	17	13.4		
Divorced/widowed	39	20.0	15	11.8		
Missing	3	1.5				
School leaving certificates						
Higher education entrance qualification	57	30.5	47	38.2		
Intermediate school certificate	87	46.5	48	39.0		
Compulsory basic secondary schooling	37	19.8	26	21.1		
No school leaving certificate	4	2.1	1	0.8		
Psychiatric disorders (ICD-10)^1^						
F10-F19	3	1.5				
F20-F29	8	4.1				
F30-F39	113	57.9				
F40-F48	24	12.3				
F60-F69	46	23.6				
F90-F98	1	0.5				
Comorbid psychiatric disorders^1^	79	40.5				
Lifetime psychiatric hospitalization	143	74.5				

F10-F19, mental and behavioral disorders due to the use of psychoactive substances; F20-F29, schizophrenia, schizotypal and delusional disorders; F30-F39, affective disorders; F40-F48, neurotic, stress-related and somatoform disorders; F60-F69, disorders of personality and behavior in adult persons; and F90-F98, behavioral and emotional disorders with onset usually occurring in childhood and adolescence.

1 Rated by attending clinician.

### Measures

#### Child Psychopathology

Patients and partners completed the German version of the *Child Behavior Checklist for ages 4–18* (CBCL/4–18; Achenbach, 1991a; Arbeitsgruppe Deutsche Child Behavior Checklist, 1998a). It is a 118-item parent-report measure assessing children’s behavioral and emotional symptoms on a three-point scale (0=“not true,” 1=“somewhat or sometimes true,” 2=“often true”). The sum scores of the two broadband subscales “internalizing problems” and “externalizing problems” were used for the analyses. Values range from 0 to 62 for internalizing and from 0 to 66 for externalizing, with higher values indicating higher levels of behavioral and emotional symptoms. The German version of the CBCL shows good reliability, as well as good factorial and convergent validity (Döpfner et al., 1994; Klasen et al., 2000). For the present study, the internal consistencies for internalizing were Cronbach’s *α*=0.90 (patient’s perspective) and 0.91 (partner’s perspective). Cronbach’s *α* for externalizing problems was 0.92 (patient’s perspective) and 0.93 (partner’s perspective).

Children’s self-reported internalizing and externalizing symptoms were assessed by the German version of the *Youth Self-Report* (YSR; Achenbach, 1991b; Arbeitsgruppe Deutsche Child Behavior Checklist, 1998b). The questionnaire is a parallel form to the CBCL and consists of 112 items rated on a three-point scale: 0=“not true,” 1=“somewhat or sometimes true,” 2=“often true.” The YSR was completed by children from the age of 12–18years. Sum scores range from 0 to 62 for internalizing and from 0 to 60 for externalizing, with higher values representing higher symptom levels. The YSR has satisfactory psychometric properties (Döpfner et al., 1995). Internal consistency was *α*=0.90 for internalizing and 0.80 for externalizing problems in the present study.

Both for the CBCL and the YSR sum scores can be transformed to T-scores with values below 60 representing the normal range, values of 60 through 63 the borderline clinical range, and values above 63 the clinical range.

#### Parental Psychopathology

Patients’ psychopathology was assessed by the German version of the *Brief Symptom Inventory* (Derogatis and Melisaratos, 1983; Franke, 2000). It is a self-report measure for adult symptomatology including 53 items. Items are measured on a five-point response scale ranging from 0 (“not at all”) to 4 (“extremely”). The General Severity Index (GSI) was used as a measure of general psychopathology (average response related to all 53 items). The GSI consists of the following nine subscales: Somatization, Obsessive–Compulsive, Interpersonal Sensitivity, Depression, Anxiety, Hostility, Phobic Anxiety, Paranoid Ideation, and Psychoticism. Franke (2000) and Geisheim et al. (2002) have reported good reliability and validity. In the present study, Cronbach’s *α* was 0.96 for the GSI.

#### Family Functioning

Family functioning was assessed by the *General Family Questionnaire* (“Allgemeiner Familienbogen,” FB-A; Cierpka and Frevert, 1994), a German questionnaire based on the “Process Model of Family Functioning” (Steinhauer et al., 1984; Cierpka, 1990). The questionnaire consists of 28 items rated on a four-point rating scale ranging from 0=“completely true” to 3=“not true at all.” Scores are obtained on the following seven subscales: Task Accomplishment, Role Behavior, Communication, Emotionality, Affectivity of Relations, Control as well as Values and Norms. The total sum score was used for the analyses reflecting the general functioning of the family with higher values indicating greater dysfunction. The total sum score of the FB-A showed values for Cronbach’s *α* of 0.93 (patients’ perspective), 0.91 (partners’ perspective), and 0.91 (children’s perspective) in the current sample.

#### Social Support

The *Oslo Social Support Scale* (OSSS; Dalgard et al., 2006) was used to assess social support of the children. It is a brief three-item instrument. The first item assesses the number of people who can provide support in the case of problems (four-point scale; 1=“none” to 4=“6 or more”), the second item concerns the perceived positive interest from other people (five-point scale; 1=“none” to 5 “a lot”), and the third item measures the availability of practical help if needed (five-point scale; 1=“very difficult” to 5=“very easy”). For the current study, the first item was changed to a five-point scale (1=“none,” 2=“1–2,” 3=“3–4,” 4=“5–6” and 5=“more than 6”) in order to adjust all response scales to the same length. The total sum score was used for the analyses ranging from 3 to 15, with higher values representing stronger social support. Kocalevent et al. (2018) confirmed the one-factor structure for the German translation of the instrument. Cronbach’s *α* was 0.69 (patient’s perspective), 0.68 (partner’s perspective), and 0.67 (children’s perspective).

### Statistical Analyses

Due to the hierarchical structure of the data (children clustered within families), we used linear multilevel models to test the hypotheses (Hox et al., 2018). First, we calculated a null model which only includes an intercept. Based on the null model, we estimated the intraclass correlation coefficient (ICC) as an indicator of the proportion of family-level variance. We then added regressors to the model (model 1) and included interaction terms between family functioning and social support (model 2). Models were compared based on the likelihood-ratio test. The maximum likelihood estimation (ML) was used for the likelihood-ratio test, the restricted maximum likelihood estimation (REML) was used for the estimation of standard errors. Explained variance was calculated according to Snijders and Bosker (1994, 2012). All continuous regressors were grand-mean centered. We conducted models to test the associations with both internalizing and externalizing problems. Analyses were performed separately for all three perspectives. To account for the fact that the three perspectives differed regarding two important characteristics (child age and single- versus two-parent families), we further conducted a series of sensitivity analyses. We reran the analyses from the patients’ and partners’ perspectives for the subsample of children aged 12years onward as well as the analyses from the patients’ and children’s perspectives for the subsample of families with a partner. All analyses were performed using IBM SPSS statistics, version 26.

## Results

### Descriptive Data

Table 2 shows means, SD, and intercorrelations of the variables from the three different rating perspectives. Regarding the level of internalizing symptoms, the majority of children and adolescents were within the borderline or clinical range: 62% (patients’ perspective), 52% (partners’ perspective), and 65% (children’s perspective). For externalizing symptoms, the proportions of children and adolescents within the borderline or clinical range were: 48% (patients’ perspective), 38% (partners’ perspective), and 23% (children’s perspective).

**Table 2:** Means, SD, and intercorrelations of study variables from the three different rating perspectives.

	*n*	*M (SD)*	1	2	3	4
Patients						
Patients’ psychopathology (BSI)	192	1.34 (0.69)	-			
Family functioning (FBA)	192	37.25 (15.22)	0.23^**^	-		
Child social support (OSSS)	285	10.52 (2.33)	−0.09	−0.32^**^	-	
Child internalizing problems (CBCL)	285	12.46 (9.25)	0.35^**^	0.28^**^	−0.30^**^	-
Child externalizing problems (CBCL)	285	12.53 (9.82)	0.26^**^	0.25^**^	−0.22^**^	0.44^**^
Partners						
Patients’ psychopathology (GSI)			-			
Family functioning (FBA)	127	30.99 (13.54)	0.07	-		
Child social support (OSSS)	200	11.12 (2.29)	−0.08	−0.23^**^	-	
Child internalizing problems (CBCL)	199	9.93 (8.66)	0.09	0.19^**^	−0.26^**^	-
Child externalizing problems (CBCL)	200	10.71 (9.70)	0.08	−0.12	−0.12	0.45^**^
Children						
Patients’ psychopathology (GSI)			-			
Family functioning (FBA)	94	33.09 (13.86)	0.10	-		
Child social support (OSSS)	94	10.57 (2.28)	−0.04	−0.33^**^	-	
Child internalizing problems (YSR)	94	16.97 (9.96)	0.17	0.31^**^	−0.19	-
Child externalizing problems (YSR)	94	11.38 (6.15)	0.26^*^	0.56^**^	−0.20^*^	0.29^**^

BSI, Brief Symptom Inventory; FBA, General Family Questionnaire; OSSS, Oslo Social Support Scale; CBCL, Child Behavior Checklist; and YSR, Youth Self-Report.

* *p*<0.05;

** *p*<0.01.

### Associations With Internalizing Problems

The results of the multilevel linear analyses for the associations with internalizing problems are displayed in Table 3.

**Table 3:** Associations with child internalizing problems from the three different rating perspectives.

	Patients^a^		Partners^b^		Children^c^	
	Model 1	Model 2	Model 1	Model 2	Model 1	Model 2
	Coeff. (95% CI)	Coeff. (95% CI)	Coeff. (95% CI)	Coeff. (95% CI)	Coeff. (95% CI)	Coeff. (95% CI)
Fixed effects						
Intercept	12.49^***^ (11.01; 13.98)	12.50^***^ (10.99; 14.01)	9.53^***^ (7.53; 11.53)	9.54^***^ (7.56; 11.53)	20.68^***^ (18.26; 23.10)	20.99^***^ (18.55; 23.44)
L1 (child level)						
Social support	−0.71^**^ (−1.18; −0.25)	−0.71^**^ (−1.18; −0.25)	−0.61^*^ (−1.18; −0.03)	−0.66^*^ (−1.23; −0.09)	−0.46 (−1.29; 0.36)	−0.55 (−1.38; 0.27)
Age	0.43^**^ (0.18; 0.67)	0.43^**^ (0.18; 0.67)	0.40^*^ (0.10; 0.70)	0.39^*^ (0.09; 0.69)	0.25 (−0.73; 1.23)	0.31 (−0.67; 1.29)
Child gender^†^	0.68 (−1.23; 2.60)	0.69 (−1.23; 2.60)	−0.07 (−2.38; 2.24)	0.10 (−2.19; 2.39)	−8.40^***^ (−12.09; −4.71)	−8.11^***^ (−11.80; −4.42)
L2 (family level)						
Patients’ psychopathology	4.36^***^ (2.78; 5.94)	4.36^***^ (2.78; 5.94)	1.50 (−0.47; 3.46)	1.18 (−0.79; 3.16)	1.49 (−1.10; 4.09)	1.33 (−1.25; 3.92)
Family functioning	0.08^*^ (0.01; 0.16)	0.08^*^ (0.01; 0.16)	0.08 (−0.02; 0.18)	0.08 (−0.02; 0.17)	0.18^**^ (0.05; 0.32)	0.18^**^ (0.05; 0.32)
Patient gender^†^	−0.87 (−3.33; 1.59)	−0.87 (−3.33; 1.60)	1.75 (−0.99; 4.49)	2.24 (−0.52; 5.00)	−1.97 (−5.98; 2.04)	−2.15 (−6.14; 1.84)
Family functioning × social support		<0.01 (−0.03; 0.03)		0.04^*^ (0.01; 0.08)		0.04 (−0.01; 0.09)
Random effects						
Variance of residuals	46.39^***^ (35.17; 61.19)	46.48^***^ (35.23; 61.31)	48.53^***^ (34.69; 67.89)	46.81^***^ (33.45; 65.50)	72.45^***^ (53.82; 97.52)	71.57^***^ (53.08; 96.51)
Variance of intercepts	20.64^**^ (10.58; 40.27)	20.81^**^ (10.70; 40.49)	20.77^*^ (8.75; 49.33)	21.34^*^ (9.33; 48.79)		
ICC	0.31	0.31	0.30	0.31		
Deviance	1989.94	1989.94	1393.63	1388.53	662.08	659.85
BIC	2040.81	2046.46	1441.27	1441.46	698.42	700.74

Linear mixed models with raw scores from the Child Behavior Checklist and Youth Self-Report. Coeff., unstandardized coefficients; ICC, intraclass correlation coefficient; BIC, Bayesian Information Criterion; grand-mean centering for all continuous regressors.

a *n*=285 children nested in 192 families.

b *n*=199 children nested in 127 families.

c *n*=94 children nested in 75 families.

† Female=0, male=1.

* *p*<0.05;

** *p*<0.01;

*** *p*<0.001.

#### Patients’ Perspective

In the null model, the ICC was *ρ*=0.37, indicating that 37% of the variance in internalizing symptoms was at the family level. In model 1 containing the regressors, the ICC was reduced to *ρ*=0.31. Compared to the null model, the model fit significantly improved as indicated by the likelihood-ratio test (*χ*^2^ (6)=72.74, *p*<0.001). Social support, child age, patients’ psychopathology, and family functioning were significantly related to child internalizing problems. Higher levels of social support were associated with a decrease in internalizing problems and higher age with an increase. On the family level, higher patients’ psychopathology and higher family dysfunction were associated with more internalizing problems. All regressors combined explained 22% of child-level variance ($R_{1}{}^{2}=0.22$) and 24% of family-level variance ($R_{2}{}^{2}=0.24$) in internalizing problems.

#### Partners’ Perspective

The null model indicated that 32% of the variance in internalizing problems was at the family level (*ρ*=0.32). After entering the regressors, the ICC was *ρ*=0.30. Compared to the null model, the model fit significantly improved (*χ*^2^ (6)=22.25, *p*=0.001). Only the child-level variables, social support and age, were significantly related to internalizing problems. Higher levels of social support were associated with a decrease in internalizing problems and higher age with an increase. Altogether, the regressors explained 8% of the variance in internalizing problems on the child level ($R_{1}{}^{2}=0.08$) and 9% of variance on the family level ($R_{2}{}^{2}=0.09$).

#### Children’s Perspective

A fixed linear regression model was applied because the estimation of the variance of the random intercept was zero. After entering the regressors, the model fit significantly improved (*χ*^2^ (6)=35.77, *p*<0.001)$C_{B}\left(v\right)=\underset{i\neq j}{\sum }\frac{G_{v}\left(i,j\right)}{G\left(i,j\right)}$ Internalizing symptoms were significantly associated with child gender and family functioning. Boys scored lower than girls on internalizing problems, and higher levels of family dysfunction were associated with more internalizing problems. The model explained 27% of variance in internalizing problems ($R^{2}=0.27$).

### Associations With Externalizing Problems

The results of the multilevel linear analyses for the associations with externalizing problems are displayed in Table 4.

**Table 4:** Associations with child externalizing problems from the three different rating perspectives.

	Patients^a^		Partners^b^		Children^c^	
	Model 1	Model 2	Model 1	Model 2	Model 1	Model 2
	Coeff. (95% CI)	Coeff. (95% CI)	Coeff. (95% CI)	Coeff. (95% CI)	Coeff. (95% CI)	Coeff. (95% CI)
Fixed effects						
Intercept	11.62^***^ (10.04; 13.19)	11.70^***^ (10.10; 13.30)	10.49^***^ (8.23; 12.75)	10.51^***^ (8.24; 12.79)	11.89^***^ (10.53; 13.26)	11.64^***^ (10.27; 13.00)
L1 (child level)						
Social support	−0.69^**^ (−1.18; −0.19)	−0.70^**^ (−1.20; −0.20)	−0.51 (−1.16; 0.14)	−0.53 (−1.18; 0.13)	0.03 (−0.44; 0.49)	0.10 (−0.36; 0.56)
Age	−0.47^**^ (−0.74; −0.21)	−0.47^***^ (−0.74; −0.21)	−0.42^*^ (−0.76; −0.07)	−0.42^*^ (−0.76; −0.07)	0.01 (−0.54; 0.57)	−0.03 (−0.58; 0.51)
Child gender^†^	3.15^**^ (1.03; 5.26)	3.17^**^ (1.06; 5.29)	1.64 (−1.01; 4.28)	1.67 (−0.98; 4.32)	1.30 (−0.78; 3.39)	1.07 (−0.99; 3.12)
L2 (family level)						
Patients’ psychopathology	2.59^**^ (0.97; 4.22)	2.60^**^ (0.97; 4.23)	0.39 (−1.81; 2.60)	0.29 (−1.95; 2.53)	1.65^*^ (0.18; 3.11)	1.78^*^ (0.34; 3.22)
Family functioning	0.12^**^ (0.04; 0.20)	0.12^**^ (0.04; 0.20)	0.08 (−0.03; 0.19)	0.08 (−0.03; 0.19)	0.24^***^ (0.17; 0.32)	0.24^***^ (0.17; 0.32)
Patient gender^†^	−2.11 (−4.65; 0.43)	−2.06 (−4.61; 0.49)	−1.46 (−4.54; 1.61)	−1.31 (−4.44; 1.82)	−3.69^**^ (−5.95; −1.42)	−3.55^**^ (−5.77; −1.32)
Family functioning × social support		0.01 (−0.02; 0.04)		0.01 (−0.03; 0.06)		−0.03^*^ (−0.06; −0.01)
Random effects						
Variance of residuals	68.46^***^ (52.40; 89.45)	68.44^***^ (52.33; 89.51)	67.16^***^ (49.02; 92.01)	66.45^***^ (48.34; 91.33)	23.16^***^ (17.20; 31.17)	22.28^***^ (16.53; 30.05)
Variance of intercepts	10.02 (2.00; 50.05)	10.24 (2.09; 50.04)	22.55^*^ (8.75; 58.10)	23.80^*^ (9.52; 59.49)		
ICC	0.13	0.13	0.25	0.26		
Deviance	2043.33	2042.97	1454.50	1454.10	554.86	550.16
BIC	2094.20	2099.49	1502.19	1507.08	591.21	591.05

Linear mixed models with raw scores from the Child Behavior Checklist and Youth Self-Report. Coeff., unstandardized coefficients; ICC, intraclass correlation coefficient; BIC, Bayesian Information Criterion; grand-mean centering for all continuous regressors.

a *n*=285 children nested in 192 families.

b *n*=200 children nested in 127 families.

c *n*=94 children nested in 75 families.

† Female=0, male=1.

* *p*<0.05;

** *p*<0.01;

*** *p*<0.001.

#### Patients’ Perspective

The null model indicated that 20% of the variance in externalizing problems was at the family level (*ρ*=0.20). After entering the regressors, the ICC was reduced to *ρ*=0.13. Compared to the null model, the model fit significantly improved as indicated by the likelihood-ratio test (*χ*^2^ (6)=63.43, *p*<0.001). Social support, child age and gender, patients’ psychopathology, and family functioning were significantly related to externalizing problems. Higher levels of social support and higher age were associated with a decrease in externalizing problems. Boys scored higher on externalizing problems. On the family level, higher patients’ psychopathology and family dysfunction were associated with more externalizing problems. The proportion of explained variance by all regressors was 19% for child-level externalizing problems ($R_{1}{}^{2}=0.19$) and 22% for family-level externalizing problems ($R_{2}{}^{2}=0.22$).

#### Partners’ Perspective

The null model indicated that 32% of the variance in externalizing problems was at the family level (*ρ*=0.32). After entering the regressors, the ICC was *ρ*=0.25. Compared to the null model, the model fit significantly improved (*χ*^2^ (6)=13.77, *p*=0.032). Only the child-level variable, age, was a significantly related to externalizing problems with higher age being associated with less externalizing problems. The model explained 5% of variance in child-level externalizing problems ($R_{1}{}^{2}=0.05$) and 8% of variance in family-level externalizing problems ($R_{2}{}^{2}=0.08$).

#### Children’s Perspective

As in the analysis on associations with internalizing problems, a fixed linear regression model was applied. Compared to the null model, the model fit significantly improved (*χ*^2^ (6)=52.47, *p*<0.001). Externalizing symptoms were significantly related to patients’ psychopathology, family functioning, and patient gender. Higher patients’ psychopathology and family dysfunction were associated with an increase in externalizing problems. In families where a father was mentally ill, children reported fewer externalizing problems than in families where a mother was mentally ill. The proportion of explained variance in externalizing problems by the regressors was 39% ($R^{2}=0.39$).

### Interaction Between Family Functioning and Social Support

**Figure 1:**
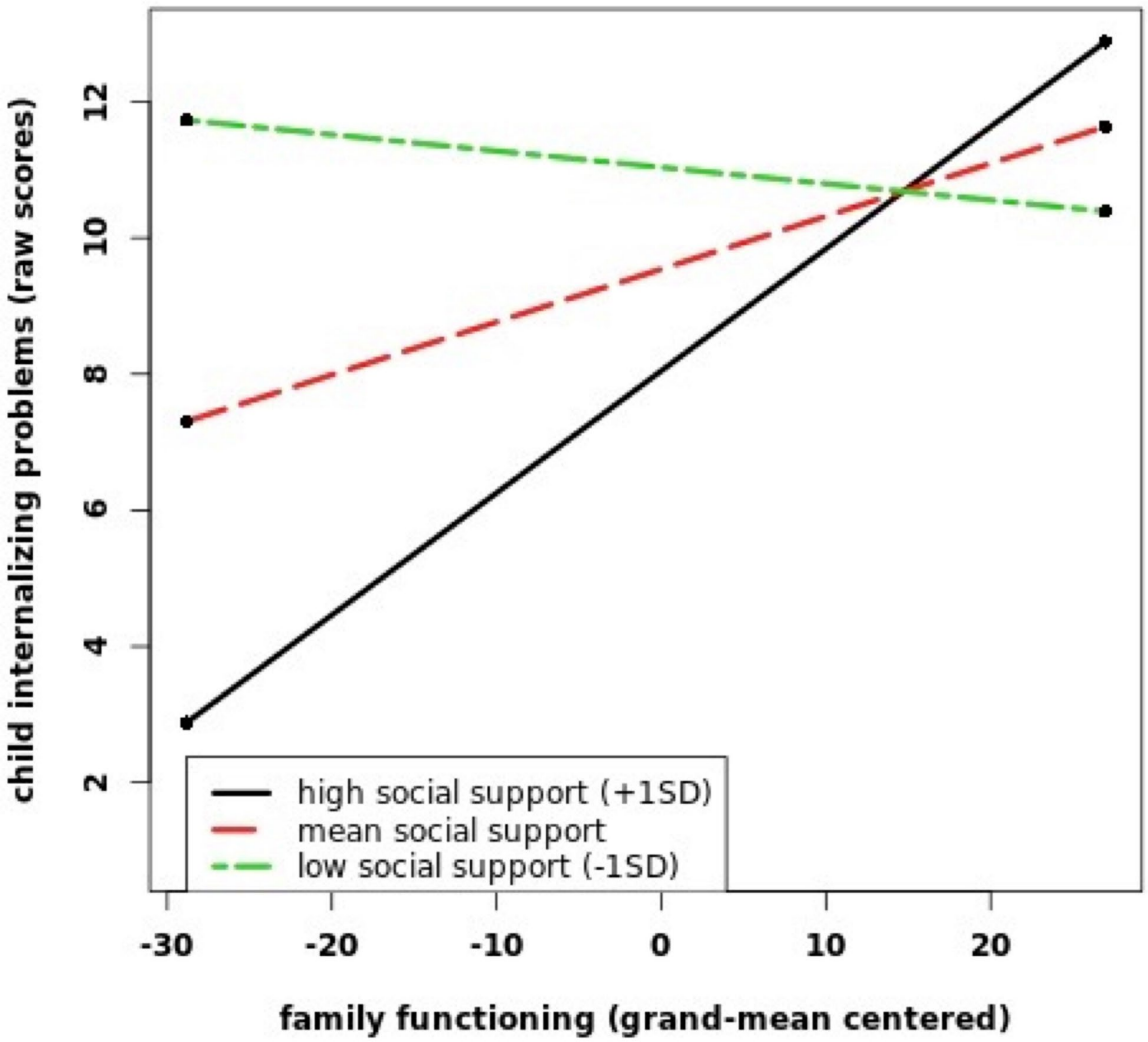
This figure shows the interaction between family functioning (FBA) and social support (OSSS) on child internalizing problems (CBCL) from the partners’ perspective. The values for FBA and OSSS are grand-mean centered.

To test, whether social support moderates the association of family functioning with child psychopathology, an interaction term between both variables was entered in a second model (see Table 3 for internalizing problems and Table 4 for externalizing problems). For the association with internalizing problems, the interaction term was non-significant from the patients’ and children’s perspectives. Also, the inclusion of the interaction term resulted in a decrease in model fit for both perspectives (for patients’ perspective: *χ*^2^ (1)<0.01, *p*=0.964; for children’s perspective: *χ*^2^ (1)=2.23, *p*=0.136). For the partners’ perspective, there was a significant interaction of family functioning by social support and an increase in model fit after inclusion of the interaction term (*χ*^2^ (1)=5.10, *p*=0.024). Simple slope analyses (Preacher et al., 2006) indicated that whereas there was no association between family dysfunction and internalizing problems for low (1*SD* below the mean, *b*=−0.02, *p*=0.721) and average levels of social support (*b*=0.08, *p*=0.113), family dysfunction was positively associated with child internalizing problems for high levels of social support (1*SD* above the mean, *b*=0.18, *p*=0.007; see Figure 1). Entering the interaction term resulted in an increase in explained variance from 8% ($R_{1}{}^{2}=0.08$) to 10% ($R_{1}{}^{2}=0.10$) on the child level and from 9% ($R_{2}{}^{2}=0.09$) to 10% ($R_{2}{}^{2}=0.10$) on the family level.

**Figure 2:**
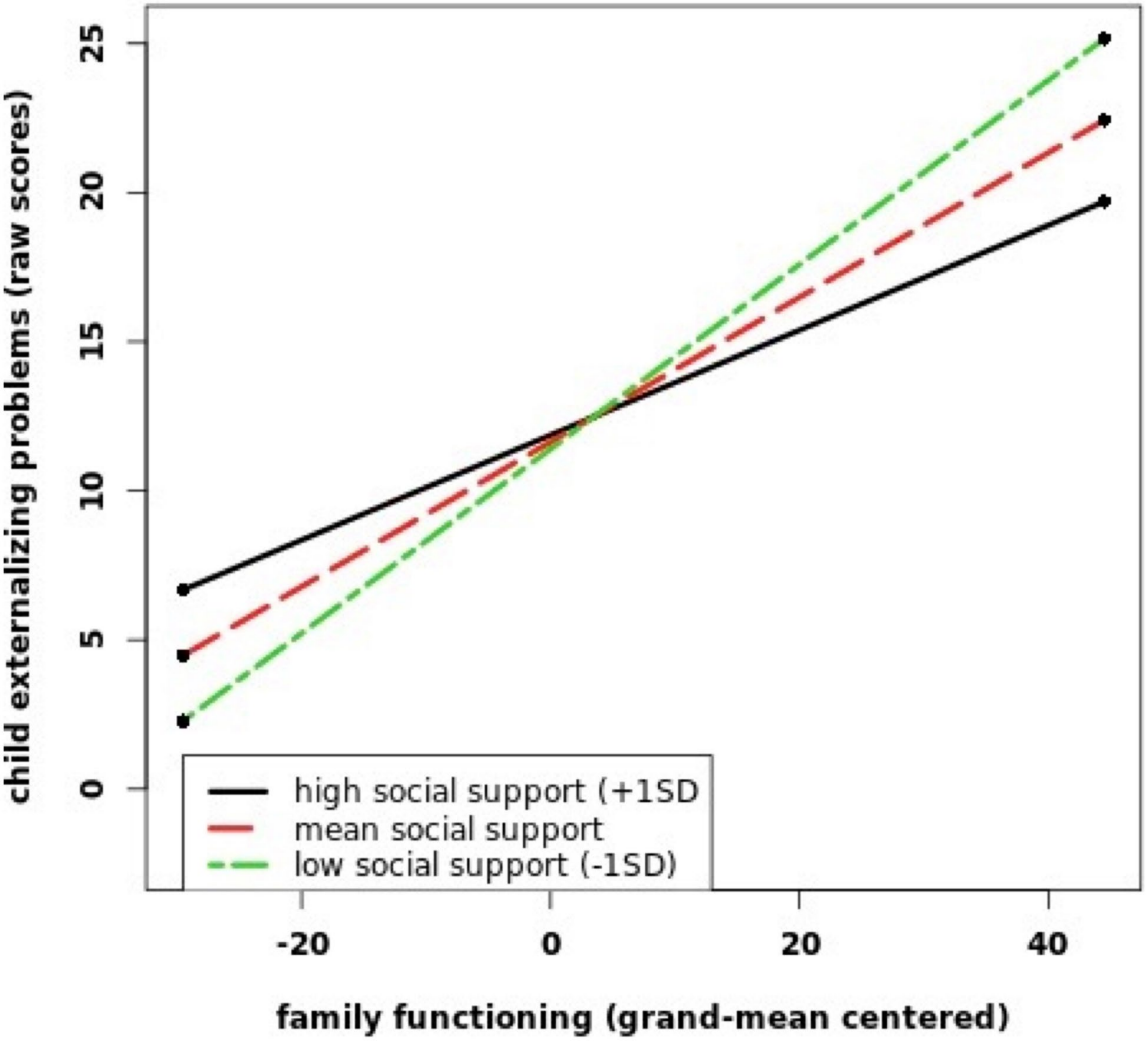
This figure shows the interaction between FBA and OSSS on child externalizing problems (YSR) from the children’s perspective. The values for FBA and OSSS are grand-mean centered.

For the association with externalizing problems, the interaction term of family functioning and social support was non-significant and led to a decrease in model fit from patients’ and partners’ perspectives (for patients’ perspective: *χ*^2^ (1)=0.36, *p*=0.547; for partner’s perspective: *χ*^2^ (1)=0.4, *p*=0.527). For the children’s perspective, there was a significant interaction effect of family functioning and social support leading to an increase in model fit (*χ*^2^ (1)=4.70, *p*=0.030). Simple slope analyses indicated that the positive relationship between family dysfunction and child externalizing problems declined as child social support increased. The effect of family dysfunction on child externalizing problems was higher for children with low (1*SD* below the mean, *b*=0.31, *p*<0.001) and average (*b*=0.24, *p*<0.001) levels of social support than for children with high levels of social support (1*SD* above the mean, *b*=0.18, *p*=0.001; see Figure 2). Entering the interaction term resulted in an increase in explained variance from 39% ($R^{2}=0.39$) to 41% ($R^{2}=0.41$).

### Sensitivity Analyses

In the sensitivity analyses, most associations remained robust. However, when reanalyzing the data from the patients’ and partners’ perspectives for children from the age of 12years onward, there were some changes from the partners’ perspective: the association between internalizing problems and social support was no longer significant (*b*=−0.66, *p*=0.225), and there was no significant interaction of family functioning by social support (*b*=0.09, *p*=0.065). When reanalyzing the data from the patients’ and children’s perspectives for families with a partner, the association between family functioning and internalizing symptoms as well as externalizing symptoms was no longer significant from the patients’ perspective (internalizing symptoms: *b*=0.04, *p*=0.436; externalizing symptoms: *b*=0.09, *p*=0.079).

## Discussion

This study tested whether family functioning and social support would be associated with children’s internalizing and externalizing problems in families affected by a parental mental disorder. Our findings indicate that both variables are important in explaining internalizing and externalizing problems, albeit with differences regarding the ratings of patients, their partners, and children.

Family functioning was related to internalizing and externalizing problems from the patients’ and children’s perspectives with higher family dysfunction being related to higher levels of child psychopathology. A positive relationship between facets of family dysfunction and child emotional and behavioral problems in children of mentally ill parents was also found by other researchers (Freed et al., 2015; Wiegand-Grefe et al., 2019). However, there was no main effect of family functioning on child behavior problems for the partners’ perception in this sample. Previous studies on family functioning did not specifically investigate a partner’s perspective in the prediction of child psychopathology. Instead, either only mentally ill parents or children’s perspectives were included (Freed et al., 2015; Plass et al., 2016; Wiegand-Grefe et al., 2019) or both parents’ ratings were conceptualized in terms of mother’s versus father’s perspectives (Burstein et al., 2012). This impedes a direct comparison of our results from the partners’ perspective with those of previous studies. Results from the sensitivity analyses might further indicate that family functioning is especially relevant for the mental health of children in single-parent families as from the patients’ perspective the association with family functioning was no longer significant when reanalyzing the data for families with a partner.

Child social support was associated with both internalizing and externalizing problems from the patients’ perspective as well as internalizing problems from the partners’ perspective. Higher social support was associated with lower levels of emotional and behavior problems in children and adolescents. The results reported here converge with those of Hoefnagels et al. (2006) in showing that social support serves as a factor predicting mental health in the offspring of mentally ill parents. However, this was not the case from the children’s perspective, which is in contrast to the findings of Hoefnagels et al. (2006) but in line with results of Plass et al. (2016). The latter also did not confirm social support as predictor for children’s self-reported psychopathology. One possible explanation for these diverging findings regards the conceptualization and assessment of social support. Findings from Hoefnagels et al. (2006) indicate that from adolescents’ perspective negative social support, meaning their perception of problematic social interactions, rather than positive social support is predictive of their mental health. We did not include this negative facet of social support in our study but rather used a more general measure of social support (Kocalevent et al., 2018). Future studies including this negative facet and taking into account specific support sources (e. g. family members, peers, and professionals) might shed more light on the role of social support for the development of psychopathology in children of mentally ill parents.

We found significant interaction effects of family functioning by social support in relation to externalizing problems from the children’s perspective. For this perspective, poor family functioning was less strongly related to externalizing problems when social support was high. This finding is in line with our hypothesis that social support would moderate the association between family functioning and child psychopathology. The result may be indicative of a buffer effect of social support against the adverse effects of family dysfunction on child mental health that has been stated in previous studies (Gauze et al., 1996; Daches et al., 2018). We also found a significant interaction between family functioning and social support in relation to internalizing problems from the partners’ perspective. From the partners’ perspective, family dysfunction was positively associated with child internalizing problems only for high levels of children’s social support. This finding is in contrast to the buffer hypothesis of social support. One possible explanation is that besides buffering stress, social support might under certain circumstances also exacerbate the adverse effects of family dysfunction on the mental health of children with mentally ill parents. Our results suggest that this might be specifically the case from the partners’ point of view. Results of a systematic review of Stiawa and Kilian (2017) showed that close relationships within the social network of children with a mentally ill parents can also be perceived as stressful and might be related to feelings of shame and fear of losing social support sources. To the best of our knowledge, we were the first to investigate this interaction effect and a replication in future studies is necessary to confirm this result – also given that the interaction effect from the partners’ perspective was not robust in sensitivity analyses.

In our analyses, we also controlled for patients’ psychopathology and sociodemographic variables. Higher patients’ psychopathology was associated with higher levels of child internalizing and externalizing problems from the patients’ perspective as well as higher levels of externalizing problems from the children’s perspective. The results regarding parental psychopathology confirm data from a range of studies. Across different parental mental illnesses, symptom severity is associated with mental health problems in the offspring (Brennan et al., 2000; Burstein et al., 2006; Beardslee et al., 2011). In our study, this association was dominantly found for the patients’ perspective rather than the partners’ and children’s perspective. This finding might be explained by the tendency of patients with a higher level of symptoms to also evaluate their children to be more symptomatic. This means that besides reflecting a positive association between patient and child symptoms, the results might also partly be explained by a potential reporting bias. Such a bias has been described in previous studies (Najman et al., 2001). Regarding child age, our results are in line with the previous research on child behavior problems in showing that the frequency of internalizing problems increases with age, whereas certain externalizing problems like aggressive behavior and attention problems decrease (Döpfner et al., 1997). Our results also corroborate previous findings that girls tend to score higher on internalizing problems, whereas boys tend to score higher on externalizing problems (Achenbach et al., 2016) and that child behavior problems are slightly more pronounced in the case of maternal mental illness (Connell and Goodman, 2002).

Adding to a large number of studies (Ford et al., 2007; Siegenthaler et al., 2012; Rasic et al., 2013; van Santvoort et al., 2015), the offspring of mentally ill parents in this study was found to show a substantial amount of behavioral and emotional problems. Particularly internalizing problems were elevated in this sample with more than 50% of children and adolescents being in the borderline or clinical range of the CBCL and YSR from all rating perspectives. These findings again highlight the at-risk status of this population.

There are several limitations of our study that must be considered when interpreting the results. One of the limitations regards the study’s cross-sectional nature. There remains some uncertainty concerning the direction of the relationship between variables. While family functioning, social support, and parental psychopathology predicted child psychopathology in this study, there might also be a reverse effect. There is increasing longitudinal research suggesting a bidirectional relationship between parent and child psychopathology (Elgar et al., 2004; Antúnez et al., 2018). Also, child psychological symptoms may have an impact on family functioning (Kashdan et al., 2004; Fleck et al., 2015) and the treatment of child mental health problems can be related to improvements in family functioning (Keeton et al., 2013). This highlights the importance of future longitudinal studies to further disentangle the associations. Furthermore, for our analyses, we used a clinical sample of families affected by parental mental illness. While we analyzed a mixed sample of parental diagnoses, the majority of patients had an affective disorder. The findings might therefore be more representative for families affected by parental affective disorders. A larger sample and a more balanced distribution of parental mental illnesses in future studies would allow to investigate whether there are differential relationships between family functioning, social support, and child mental health depending on specific groups of parental illnesses. To improve diagnostic accuracy, future studies should also include standardized diagnostic interviews for the assessment of parental diagnoses, like the Structured Clinical Interview for DSM (SCID; First et al., 2016). Finally, the current sample was drawn from a randomized controlled trial evaluating a family-based intervention and families who consent to participate in such trials may differ from those who decline to participate.

Besides the limitations, there are also strengths of our study. Data were obtained from multiple informants. We included both maternal and paternal mental illnesses. While a lot of past research focused on maternal mental illness only, recent studies have shown that also fathers play essential roles for the development of psychopathology in their children (Ramchandani and Psychogiou, 2009). For our study, we concentrated on potentially modifiable variables related to child psychopathology, which leads to some clinical implications.

Our results suggest that family functioning and social support serve as relevant risk and protective factors in the context of parental mental illness. Researchers should further improve the knowledge based on malleable risk and protective factors related to the mental health of children with mentally ill parents. Moreover, progress in the development and long-term evaluation of preventive programs is needed (Thanhauser et al., 2017). There are a range of family-based interventions that have shown significant improvements in the mental health of children (Siegenthaler et al., 2012; Thanhauser et al., 2017) and family functioning (Beardslee et al., 2007). Besides family interventions, social support groups for children of mentally ill parents allow for mutual support and have shown to be effective in improving the use of social support sources (Van Santvoort et al., 2014).

However, these interventions are still offered only to a small range of families and implementation barriers need to be overcome to offer them to a larger number of families in the future (Krumm et al., 2013). One fundamental requirement is a change in organizational policies and procedures toward a more family-focused approach (Maybery and Reupert, 2009; Grove et al., 2017). Professionals working with adult psychiatric patients need to systematically assess whether their patients are parents and take children’s and family members’ needs into account (Krumm et al., 2013). Further, health care professionals should be provided with more knowledge related to family functioning and social support resources to detect children at risk and provide them with the appropriate support.

## Data Availability Statement

The raw data supporting the conclusions of this article will be made available by the authors, without undue reservation.

## Ethics Statement

The studies involving human participants were reviewed and approved by Ethics Committee of the Chamber of Physicians in Hamburg, Germany under the number PV4744, 05.08.2014. Written informed consent to participate in this study was provided by the participants’ legal guardian/next of kin.

## Author Contributions

MSe and SW-G were responsible for the conceptualization of the present study. MSe performed the formal analysis with methodological support provided by AD and CB. MSe wrote the manuscript, and CB, AD, MB, and MSt revised and edited the manuscript. Data were provided by the CHIMPS multicenter study group with SW being the principal researcher. SW-G, RK, ML, and KW were significantly involved in the conception of the CHIMPS multicenter study. KW and AD provided methodological support for the project. SW-G, BA, and KW managed the project in the coordinating study center. RK, MSt, and SW were responsible for the realization of the project at their study sites. MB was responsible for coordinating data curation. All authors have read and agreed to the final version of the manuscript.

## Funding

Funding for this study was provided by the German Federal Ministry of Education and Research (BMBF; 01GY1337).

## Conflict of Interest

The authors declare that the research was conducted in the absence of any commercial or financial relationships that could be construed as a potential conflict of interest.

## Publisher’s Note

All claims expressed in this article are solely those of the authors and do not necessarily represent those of their affiliated organizations, or those of the publisher, the editors and the reviewers. Any product that may be evaluated in this article, or claim that may be made by its manufacturer, is not guaranteed or endorsed by the publisher.
